# Detection of atrial fibrillation using a nonlinear Lorenz Scattergram and deep learning in primary care

**DOI:** 10.1186/s12875-024-02407-3

**Published:** 2024-07-20

**Authors:** Yi Yao, Yu Jia, Miaomiao Wu, Songzhu Wang, Haiqi Song, Xiang Fang, Xiaoyang Liao, Dongze Li, Qian Zhao

**Affiliations:** 1 General Practice Ward/International Medical Center Ward, General Practice Medical Center, West China Hospital, Sichuan University, Chengdu, China; 2 Teaching&Research Section, General Practice Medical Center, West China Hospital, Sichuan University, Chengdu, China; 3 General Practice Medical Center and General Practice Research Institute, West China Hospital, Sichuan University, Chengdu, China; 4 Department of Emergency Medicine and Laboratory of Emergency Medicine, West China Hospital, Sichuan University, Chengdu, China

**Keywords:** Atrial fibrillation, Computer-assisted diagnosis model, Convolutional neural network, Lorenz scattergram, Primary care

## Abstract

**Background:**

Atrial fibrillation (AF) is highly correlated with heart failure, stroke and death. Screening increases AF detection and facilitates the early adoption of comprehensive intervention. Long-term wearable devices have become increasingly popular for AF screening in primary care. However, interpreting data obtained by long-term wearable ECG devices is a problem in primary care. To diagnose the disease quickly and accurately, we aimed to build AF episode detection model based on a nonlinear Lorenz scattergram (LS) and deep learning.

**Methods:**

The MIT-BIH Normal Sinus Rhythm Database, MIT-BIH Arrhythmia Database and the Long-Term AF Database were extracted to construct the MIT-BIH Ambulatory Electrocardiograph (MIT-BIH AE) dataset. We converted the long-term ECG into a two-dimensional LSs. The LSs from MIT-BIH AE dataset was randomly divided into training and internal validation sets in a 9:1 ratio, which was used to develop and internally validated model. We built a MOBILE-SCREEN-AF (MS-AF) dataset from a single-lead wearable ECG device in primary care for external validation. Performance was quantified using a confusion matrix and standard classification metrics.

**Results:**

During the evaluation of model performance based on the LS, the sensitivity, specificity and accuracy of the model in diagnosing AF were 0.992, 0.973, and 0.983 in the internal validation set respectively. In the external validation set, these metrics were 0.989, 0.956, and 0.967, respectively. Furthermore, when evaluating the model’s performance based on ECG records in the MS-AF dataset, the sensitivity, specificity and accuracy of model diagnosis paroxysmal AF were 1.000, 0.870 and 0.876 respectively, and 0.927, 1.000 and 0.973 for the persistent AF.

**Conclusions:**

The model based on the nonlinear LS and deep learning has high accuracy, making it promising for AF screening in primary care. It has potential for generalization and practical application.

**Supplementary Information:**

The online version contains supplementary material available at 10.1186/s12875-024-02407-3.

## Background

Atrial fibrillation (AF) is one of the world’s most challenging cardiovascular diseases of the 21st century and is strongly associated with stroke, heart failure and death [1, 2]. However, approximately 30% of AF patients can be asymptomatic or subclinical [3]. Although the relationship between AF screening and clinical outcomes is controversial [4]. Various devices can improve the detection of atrial fibrillation and facilitate the early adoption of comprehensive intervention, to reduce complications [5]. In recent years, wearable technologies for AF screening have been rapidly developing, with advantages such as home use, portability and long-term monitoring [1]. There are two types of wearable ECG devices, one is the device that monitors the electrical activity of the heart, and the other is the device based on photoplethysmography (PPG) [6]. However, the diagnosis of arrhythmias using PPG devices needs to be confirmed by ECG, which limits their practical application in clinical practice. Therefore, the devices that obtain ECG signals has become the focus of this study. A randomized clinical trial showed that long-term wearable ECG devices increased AF detection in the community by 4.1% [7].

Interpreting long-term ECG data obtained by wearable devices is time-consuming, which restrict the rapid diagnosis of AF by general practitioners in primary care [8, 9]. In recent years, there has been a growing number of machine learning models that have been developed to enable rapid diagnosis of long-term ECG [10]. These models are constructed from the ECG characteristics of atrial and ventricular electrical activity [11–13]. Santala et al. showed that an automated artificial intelligence arrhythmia detection achieved a sensitivity of 100% and a specificity of 95.4% for detection of AF from 24-hour single-lead heart belt recordings in a clinical setting [14]. However, the study did not provide specific information about the intelligent model used, making it difficult to replicate and validate. Hartikainen et al. reported that COSEn and AFEvidence algorithms was achieved with sensitivity of 95.3%/96.3% and specificity of 95.5/98.2% for detection of AF from chest strap [15]. However, These models use traditional shallow machine learning algorithms, which require manual feature extraction and are easily overfit [16]. Deep learning, including convolutional neural networks (CNNs) and recurrent neural networks (RNNs), is an emerging branch of machine learning. It uses an end-to-end learning mechanism to achieve direct output prediction without human intervention [17]. Several studies have found that deep learning can generally perform better than traditional ECG modeling algorithms [18–20]. A study published in *The Lancet* found that CNNs can discover ECG features that are difficult to discern by the human eye and demonstrated the advantages of CNNs in ECG image analysis [21].

However, the heart is essentially a nonlinear chaotic system, and the current research did not consider the nonlinear characteristics. The Lorenz scattergram (LS) is an ECG analysis technique based on chaos theory, which converts traditional linear ECG into a nonlinear two-dimensional image. In LS, homogenous heartbeats gather together to form an “attractor“ [22]. The attractor shows a characteristic pattern and is used for arrhythmia diagnosis [22]. In recent years, various diagnostic models based on an LS have emerged that are only constructed by shallow machine learning algorithms [13, 23, 24]. Kisohara et al. [25] used CNNs and a LS to establish an AF diagnostic model and found that the optimal segment window length for detecting AF with 32 × 32-pixel LS is 85 beats. However, this study only included sinus rhythm and persistent AF in the model training and did not include paroxysmal AF or other arrhythmias. The generalization ability of this model in the clinic weakened. Therefore, it is necessary to establish a deep learning-based model that can diagnose persistent and paroxysmal AF on long-term ECG and validate it in primary care.

## Methods

### ECG dataset

This is a cross-sectional study. We extracted the MIT-BIH Normal Sinus Rhythm Database [26], the MIT-BIH Arrhythmia Database [27] and the Long-Term AF Database [28] from PhysioNet and constructed the MIT-BIH Ambulatory Electrocardiograph (MIT-BIH AE) dataset for training and internal validating the diagnosis model. The AF records were labeled as AF. Sinus rhythm and other arrhythmias were labeled as non-AF.

We built a MOBILE-SCREEN-AF (MS-AF) dataset, which came from a single-lead wearable ECG device in a community hospital in Chengdu, Sichuan and was used for external validation. This hospital is located in the suburbs of Chengdu. It has jurisdiction over 16 communities and serves a population of 138,700. We prospectively enrolled participants. The inclusion criteria for this study were as follows: (i) patients with one or more of the following 7 high-risk factors for atrial fibrillation: age ≥ 65 years, hypertension, diabetes, obesity (Chinese standard, BMI ≥ 28 kg/m^2^ [29]), smoking, drinking, and myocardial infarction; and (ii) Voluntary participation and provision of health examination information. The exclusion criteria were as follows: (i) patients with a pacemaker or ICD; (ii) patients with a history of severe skin allergy (Severe skin allergy is defined as the presence of one or more symptoms, including localized or systemic skin breakdown, loss of consciousness, decreased blood pressure, shortness of breath, fast and weak pulse, nausea or vomiting.); and (iii) patients who were expected to wear the device for less than 24 h. For participants who provided consent, we equipped them with wearable ECG devices. The device is a patch that is used with wet electrodes. The device, provided by Chengdu Synwing Technology Co., Ltd. (Medical device registration number: 20,212,070,096), is capable of continuous monitoring for up to 72 h. The device is showed in Appendix Fig. 1. The positions and diagnostic labels of R waves were first performed by a computer software. ECG technician cleaned up single-lead ECG data. The cleanup process consists of three parts. Firstly, he assessed whether the actual monitoring time is equal to or greater than 24 h. Secondly, he evaluated the quality of the ECG waveforms, which included two steps. The first step was noise reduction processing of the ECG, based on the high-pass filtering, low-pass filtering and band-stop filtering techniques. The second step was to assess whether there are still unidentifiable ECG segments in the denoised ECG. An unidentifiable ECG segment is defined as a segment in which the precise location of the R wave cannot be accurately determined. If the proportion of unidentifiable ECG segments exceeds 10% of the actual monitoring time, the record was marked as low quality and was excluded. Third, he evaluated whether the computer software correctly labeled the R-wave location and diagnostic label (AF or non-AF). And corrected any erroneous labels. Subsequently, a cardiologist and electrocardiologist from a tertiary hospital independently reviewed the diagnostic marks of R waves, and inconsistencies were determined after discussion. The AF records were labeled as AF. Sinus rhythm and other arrhythmias were labeled as non-AF. This study was approved by the Ethics Committee of West China Hospital of Sichuan University (2018 − 454).

### Lorenz Scattergram

**Fig. 1:**
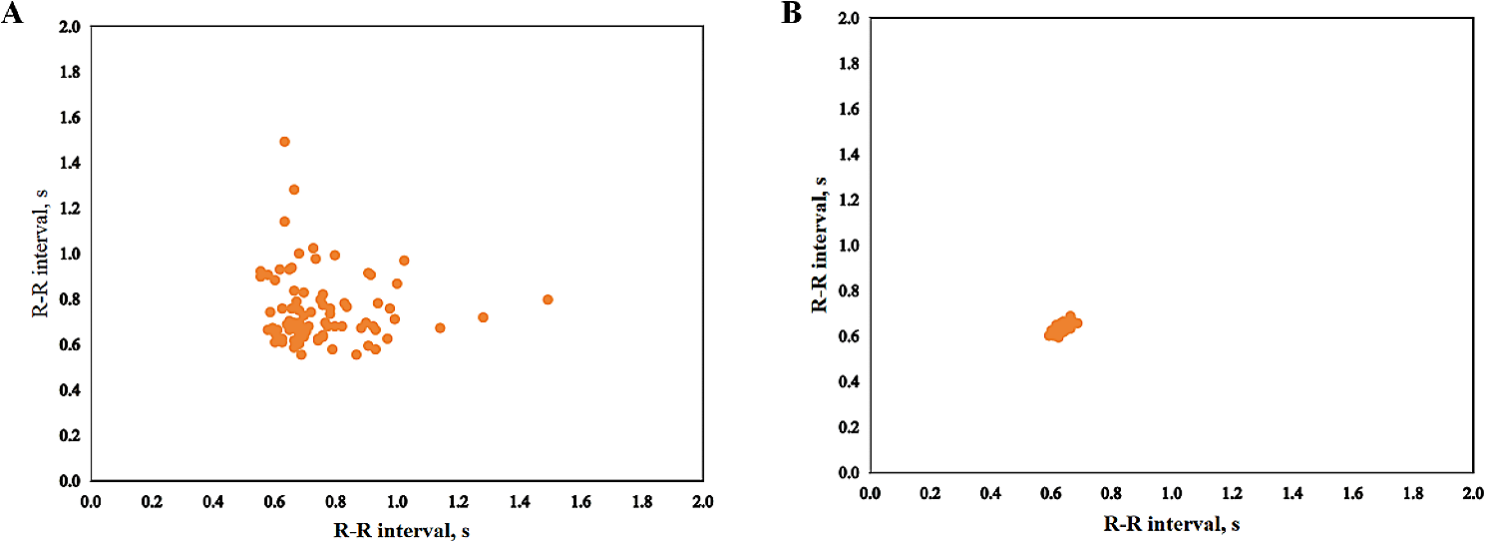
Lorenz scattergram with 85 R waves. **A** LS of AF. **B** LS of non AF (sinus rhythm)

The LS was constructed from ECG segments by determining a point (RRn, RRn + 1) in the two-dimensional coordinate system (RRn, RRn + 1 is the adjacent RR interval on the ECG). The RR interval was iterated successively to form an LS [22]. The LS preparation process was as follows: (1) The ECG recording R-wave position and diagnostic markers were converted into a text file with the suffix.rri3. (2) The text files containing R-wave positions and diagnostic markers were converted into TFRecord data streams. TFRecord is a binary file provided by TensorFlow to store data, which is convenient for copying, moving or cutting data without requiring another marker file. (3) The TFRecord data stream was continuously cut into units of 85 R waves to obtain data segments. (4) The TFRecord data segments were converted into an LS with a resolution of 32 × 32. (5) The LS was considered to be AF if the R waves of 30 consecutive seconds or more were marked as AF in the 85 R waves; otherwise, it was non-AF. Figure 1 shows an LS of AF and non AF (sinus rhythm) with 85 R waves.

We converted the ECG into a two-dimensional LS according to the aforementioned procedure. Data augmentation was used to expand the sample size for the MIT-BIH AE dataset. We used 85 R waves as the window width and 9 R waves as the step length and continuously cut the TFRecord data stream. LSs from the MIT-BIH AE dataset were randomly divided into training and validation sets in a 9:1 ratio. Data augmentation was not used in the MS-AF dataset, and LSs from the MS-AF dataset constituted the external validation set.

### Deep learning model

The CNN in this study comprised an input layer, convolutional layer, pooling layer, fully connected layer and output layer. The detailed process of model development was shown in the supplementary information. And the structure of the CNN in this study was described in Appendix Table 1. During the training and validation phases, we noticed no significant improvement in accuracy and loss value after 20 epochs. We used 20 epochs to train the model. The accuracy and loss function value of the diagnostic model of each epoch in CNN training and validation were shown in Appendix Fig. 2 and Appendix Fig. 3. We further visually analyzed the convolution kernel of the three convolution layers. The image features of the convolution kernel of the convolutional layers of the model were shown in Appendix Fig. 4, Appendix Fig. 5 and Appendix Fig. 6.

### Evaluation metrics

We selected sensitivity (Sen), specificity (Spe), accuracy (Acc), positive predictive value (PPV), negative predictive value (NPV), positive likelihood ratio (+ LR), negative likelihood ratio (-LR), receiver operating characteristic curve (ROC) and area under the curve (AUC) to evaluate model diagnostic performance [30, 31].

The performance evaluation of the model was divided into two levels. In the first level, based on the LS with 85 R waves, the predicted classification of the model was compared with the real classification of the LS to evaluate the model performance. The second level was based on long-range ECG records. Each ECG record generated several LSs with 85 R waves through sequential data cutting. The model predicted the diagnosis of ECG records according to the LS prediction results. The prediction results of the ECG record of the model were compared with the real diagnosis results of the ECG records to evaluate the model performance.

### Statistical analysis

Data were analyzed by SPSS 26.0. If the measurement data met the normal distribution, they were described by the mean±standard deviation. We used the median and quartile if the normal distribution was not satisfied. Enumeration data were described by frequency and percentage. The computer configuration was as follows: MacBook Air (2017), 1.8 GHz dual-core Intel Core i5 processor, 8 GB 1600 MHz DDR3 memory and an Intel HD Graphics 6000 1536 MB graphics card. GPU: NVIDIA TESLA P100. We used Python (version 3.8.8) for programming. The Keras software package was used for constructing and optimizing the CNN, *sklearn.metrics* was used to create confusion matrices, the *roc_curve* function was used to create ROC and calculate the AUC. The optimal threshold of the model was determined by the maximum Youdan index. The *np.argmax* function was used to find the maximum Youdan index and to determine the ROC cutoff value.

## Results

### Baseline characteristics of datasets

The MIT-BIH Normal Sinus Rhythm Database includes 18 ECG records. The MIT-BIH Arrhythmia Database includes 48 ECG records, and the Long-Term AF Database includes 84 ECG records. In the MS-AF dataset, a total of 138 patients used single-lead wearable ECG devices between September 8 and December 31, 2021. Twenty-five patients were excluded, 8 patients did not have complete health information, 3 patients had an actual measurement time of less than 24 h, and 14 patients (10.77%) had poor-quality ECG data. Finally, 113 patients were included in the analysis. The characteristics of the ECG datasets are summarized in Table 1.

**Table 1:** Baseline characteristics of the ECG datasets

Variable	MIT-BIH AE dataset			MS-AF dataset(*n* = 113)
	MIT-BIH Normal Sinus Rhythm Database (*n* = 18)	MIT-BIH Arrhythmia Database (*n* = 48)	Long-Term AF Database (*n* = 84)	
**Sex, n (%)**				
Male	5 (27.78)	26 (54.17)	-	55 (48.67)
Female	13 (72.22)	22 (45.83)	-	58 (51.33)
Age, (Mean ± SD)/M(IQR), years	34.33 ± 8.44	67.00(53.75,76.25)	-	71.0(65.00,77.00)
Duration of ECG Record, (Mean ± SD)/M(IQR), h	24.31 ± 0.86	0.50 ± 0.00	24.00(23.78,24.58)	25.08 (23.00)
Sinus Rhythm, n (%)	18(100.00)	44(91.67)	53(63.10)	72 (63.72)
**Atrial Fibrillation, n (%)**				
Paroxysmal Atrial Fibrillation	0(0.00)	5(10.42)	52(61.90)	5 (4.42)
Persistent Atrial Fibrillation	0(0.00)	3(6.25)	31(36.90)	41 (36.23)
**Other Arrhythmias, n (%)**				
Pacemaker Rhythm	-	3 (6.25)	-	0(0.00)
Supraventricular Premature Beats	15(83.33)	27(56.25)	46(54.76)	72 (63.72)
Supraventricular Tachycardia	-	7(14.58)	45(53.57)	45 (39.82)
Paroxysmal Atrial Flutter	-	2 (4.17)	-	2 (1.77)
Premature Ventricular Contractions	5(27.78)	37(77.08)	24(28.57)	91 (81.42)
Ventricular Tachycardia	-	13(27.08)	34(40.48)	9 (7.96)
Ventricular Flutter	-	1(2.08)	-	0 (0.00)

- indicates that PhysioNet did not provide the data

### Characteristics of the lorenz scattergram

A total of 1,345,849 LSs were obtained from the MIT-BIH AE dataset. The training set comprised 1,211,264 LSs, and the internal validation set comprised 134,585 LSs. In the training set, there were 575,820 cases (47.54%) of AF and 635,444 cases (52.46%) of non-AF. In the internal validation set, 66,949 cases (49.74%) of LSs were AF, and 67,636 cases (50.26%) were non-AF. A total of 207,540 LSs were transformed from the MS-AF dataset to form the external validation set. There were 67,263 cases (32.41%) of AF and 140,277 cases (67.59%) of non-AF.

### The cutoff value of the model

**Fig. 2:**
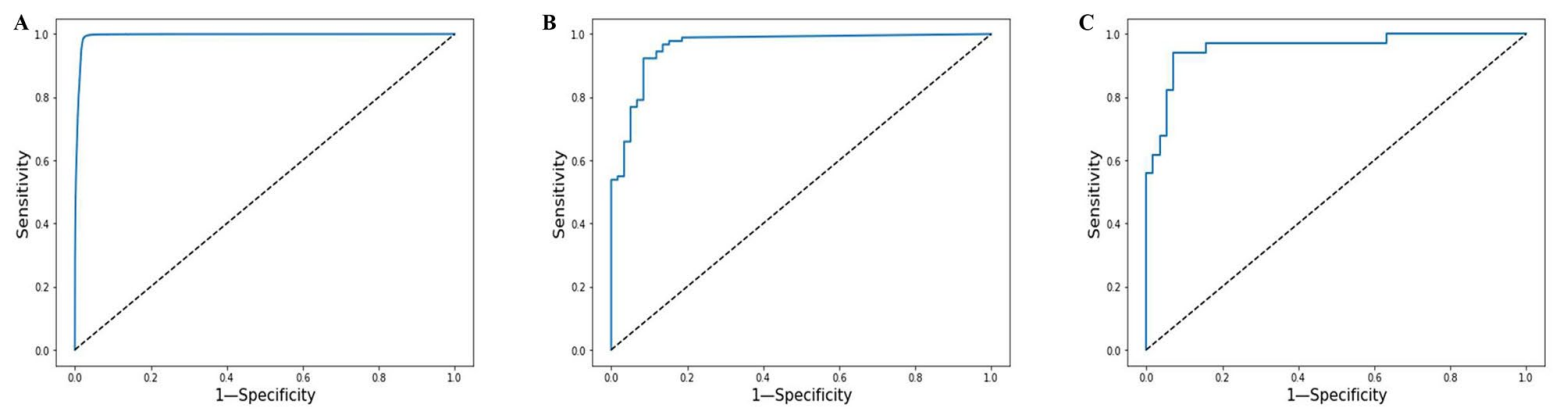
Receiver operating characteristic curves. **A** ROC of the diagnostic model in all LSs from the training and internal validation set. **B** ROC of the diagnostic model in the MIT-BIH AE dataset of ECG records. **C** ROC curves of the diagnostic model in the MIT-BIH AE dataset of AF records

The first cutoff value of the model was to distinguish whether the LS was AF or not. The ROC curves of the model for diagnosing AF in all LSs from the training and internal validation sets were shown in Fig. 2(A). The cutoff value of the model for diagnosing whether LS was AF or non-AF was 0.610. A probability value ≥ 0.610 predicted AF, and < 0.610 predicted non-AF. The second cutoff value was to distinguish whether a single-lead ECG record was AF or not. The ROC curves of the model for diagnosing AF in the MIT-BIH AE dataset were shown in Fig. 2(B). The cutoff value of the model for diagnosing whether the ECG record was AF was 0.007. A probability value ≥ 0.007 predicted AF, and < 0.007 predicted non-AF. The third cutoff value was to distinguish whether a single-lead ECG recording is persistent AF or paroxysmal AF. The ROC of the model for diagnosing paroxysmal AF in the MIT-BIH AE dataset of AF records is shown in Fig. 2(C). The cutoff value of the model for diagnosing whether the ECG record was paroxysmal AF or persistent AF was 0.948, 0.007 ≤ probability value < 0.948 predicts paroxysmal AF, and ≥ 0.948 predicts persistent AF.

### Performance evaluation in the lorenz scattergram

**Fig. 3:**
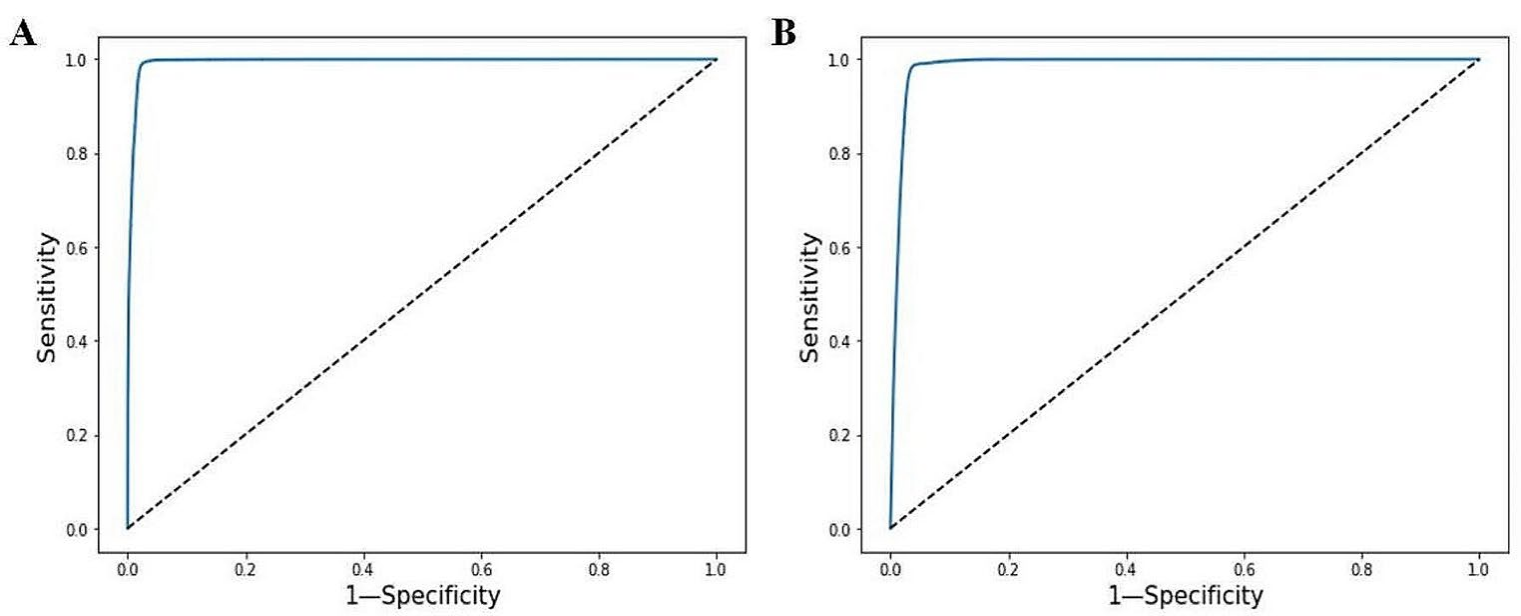
Receiver operating characteristic curves. **A** ROC of the model for diagnosing AF in the internal validation set. **B** ROC of the model for diagnosing AF in the external validation set

The confusion matrix for diagnostic model in the internal and external validation sets was shown in Table 2. In the internal validation set, the sensitivity of the model for diagnosing AF was 0.992, the specificity was 0.973, and the accuracy was 0.983. In the external validation set, the sensitivity of the model for diagnosing AF was 0.989, the specificity was 0.956, and the accuracy was 0.967. Table 3 showed the performance of the model in the internal and external validation sets. The ROC curves of the model for diagnosing AF in the internal and external validation sets were shown in Fig. 3.

**Table 2:** The confusion matrix for the model in the internal and external validation sets

		Ture label			
		Internal validation set		External validation set	
		AF	non-AF	AF	non-AF
Predicted label	AF	66,509	1,791	66,492	6,128
	non-AF	514	65,771	771	134,099

AF: Atrial fibrillation, non-AF: nonatrial fibrillation

**Table 3:** Performance of the model in the internal and external validation sets

	Sen	Spe	Acc	PPV	NPV	+LR	-LR	AUC
Internal validation set	0.992	0.973	0.983	0.974	0.992	37.434	0.008	0.995
External validation set	0.989	0.956	0.967	0.916	0.994	22.621	0.012	0.987

Sen: sensitivity, Spe: specificity, Acc: accuracy, PPV: positive predictive value, NPV: negative predictive value, +LR: positive likelihood ratio, -LR: negative likelihood ratio, AUC: area under the curve

### Performance evaluation in ECG records

In the 113 ECG records from the MS-AF dataset. The sensitivity of the model diagnosis of paroxysmal AF was 1.000, the specificity was 0.870, and the accuracy was 0.876. The sensitivity of the model diagnosis of persistent AF was 0.927, the specificity was 1.000, and the accuracy was 0.973. Table 4 shows the performance of the model in ECG records of the MS-AF dataset.

**Table 4:** Performance of the model in ECG records of the MS-AF dataset

	Sen	Spe	Acc	PPV	NPV	+LR	-LR
Non-AF	0.836	1.000	0.903	1.000	0.807	NA	0.171
Paroxysmal AF	1.000	0.870	0.876	0.263	1.000	7.692	0.000
Persistent AF	0.927	1.000	0.973	1.000	0.960	NA	0.091

Non-AF: non atrial fibrillation, Sen: sensitivity, Spe: specificity, Acc: accuracy, PPV: positive predictive value, NPV: negative predictive value, +LR: positive likelihood ratio, -LR: negative likelihood ratio, AUC: area under the curve, NA: Not applicable

## Discussion

In this study, we developed a deep learning model for detecting AF episodes based on nonlinear LS. The proposed model was external validated in a clinical ECG dataset. We found that the sensitivity, specificity and accuracy of the model in diagnosing paroxysmal AF were 100.0%, 87.0% and 87.6%, respectively, in long-term ECG records. The sensitivity, specificity and accuracy were 92.7%, 100.0% and 97.3%, respectively, for diagnosing persistent AF in long-term ECG records.

In recent years, various machine learning algorithms have emerged for diagnosing AF, including shallow machine learning algorithms and deep learning algorithms [32]. Shallow machine learning algorithms need to manually extract features, which are prone to overfitting. Deep learning algorithms automatically extract features based on raw data, which is conducive to recognizing of high-dimensional features [32]. Deep learning algorithms include CNN, long short-term memory(LSTM) and hybrid deep models(CNN-LSTM) [16]. Xia et al. [33] first proposed a CNN model for diagnosing AF, converting the 5-second ECG segment into a two-dimensional matrix by short-term Fourier transform and stationary wavelet transform and inputting a two-dimensional matrix into CNN to build a model. The model’s sensitivity and specificity for diagnosing AF were 98.34/98.79% and 97.87/98.24%, respectively, in the MIT-BIH AF database. However, this model was not validated in a clinic, and nonlinear ECG characteristics were not considered [34, 35]. Mittal et al. used a 2-part(a QRS complex detector and an AF detector) deep neural network filter to identify AF episodes in ECG data from implantable loop recorder [36]. This study demonstrated deep neural network can filter up to 66% of all false-positive AF events, and reduce the burden on clinicians. However, the model was fed with PDF reports (low resolution) instead of ECG signals, which affected the performance of the model. Another study used LSTM [37] to diagnose AF and achieved a sensitivity and specificity of 99.8% and 99.6% in MIT-BIH Atrial Fibrillation Database. Andersen et al. [19] used a CNN-LSTM for AF diagnosis, which achieved a sensitivity and specificity of 98.98% and 96.95%, respectively in Physionet dataset (MIT-BIH AF Database, the MIT-BIH Arrhythmia Database and the MIT-BIH NSR Database). The diagnostic performance of the LSTM and CNN-LSTM models is similar to that of the CNN model, but model training has a heavy calculation burden and high time consumption. In our study, we used a CNN to build the model, which has a simple structure, short training time, simple GPU configuration, good performance in primary care.

The common AF diagnosis model was mainly constructed according to the characteristics of atrial fibrillation f waves and irregular RR intervals [11, 38–40]. Nuryani et al. [11] extracted the number of P/f-wave peaks and the P/f-wave width and used the fuzzy inference system to construct a diagnostic model. The sensitivity, specificity, and accuracy of diagnosing AF were 77.89%, 60.40%, and 75.90%, respectively in MIT-BIH arrhythmia dataset. The diagnostic performance was low because the low peak value of f wave is difficult to extract, while the clinical validation process was lacking. Logan et al. [41] extracted the RR interval variance in the 10-second ECG segment to build an AF detection algorithm, with a sensitivity of 96.0% and a specificity of 89.0% in the MIT-BIH AF database. This model’s performance has been improved, but the model was also not validated in the clinic. Du et al. [38] combined the three features of “average number of f waves in TQ interval”, “maximum difference of RR interval” and “standard deviation of RR interval” of 6-second ECG segments for AF diagnosis. The accuracy of classifying AF or non-AF was 93.67%, and the specificity was 94.13% in the multiple MIT-BIH databases. However, the model was only validated in the open source database, and there might be overfitting and a lack of clinical verification. Importantly, none of the above models consider the nonlinear characteristics of ECG data.

Heart rhythm is essentially a nonlinear chaotic system. Advances in nonlinear analysis techniques of electrical signal processing could lead to a better arrhythmia diagnosis [42]. The LS is a nonlinear analytical technique based on chaos theory that can fully reflect the nonlinear heart characteristics and transform the traditional linear electrocardiogram into a nonlinear two-dimensional image [22]. Based on the LS, other scattergram analysis techniques have appeared in recent years. Examples include the RR difference scattergram [43], RdR scattergram [44] and three-dimensional scattergram [45]. Lian et al. [46] established a diagnostic model based on the RdR scattergram of different RR intervals (32, 64, and 128 RR intervals), which yielded excellent sensitivity and specificity for window sizes of 32 (94.4% and 92.6%, respectively), 64 (95.8% and 94.3%), and 128 (95.9% and 95.4%) in 4 PhysioNet databases(MIT-BIH AF database, MIT-BIH arrhythmia database, MIT-BIH normal sinus rhythm database, and normal sinus rhythm RR interval database). However, it does not use real-world ECG data for verification, nor does it consider the diagnostic value of the model in long-range ECG recordings. Mark Lown et al. [13] established a AF diagnostic model based on the decorrelated Lorenz plot of 60 consecutive RR intervals and support vector machine. The sensitivity and specificity of the model were 100.0% and 97.6% in the validation data from heart rate monitor device (Polar-H7). However, the model only considered its diagnostic performance in short-range ECG (60 RR intervals). Its diagnostic performance in long range ECG was not further verified. On the other hand, the validation data were collected in a relaxed state without an interference signal. The performance of model was limited in long-term ECG data because of long-term ECGs with rich noise and baseline drift [47]. Single-lead wearable ECG devices lack supplementary information from other leads, and are more susceptible to noise and baseline drift, resulting in poor quality. A study indicated that wearable devices based on PPG signals had poor quality, resulting in an inability to determine diagnoses in as high as 32.2% of the data [48]. Another study found that approximately 20% of data from the heart belt ECG could not be interpreted because of poor quality [14]. In our study, the proportion of low-quality ECG data was 10.77%. This percentage is lower than other studies. This may be attributed to differences in the evaluation methods for data quality. Further research is warranted.

Our model was established based on CNN and LS adequately considered the nonlinear ECG data characteristics and achieved good performance in long-range ECG in the real world. The sensitivity of the model is high, which makes it unlikely that AF was missed. It can be used for screening in community settings. In addition, the specificity of the model is high and false positive is not easy to occur. The high performance of the model allows for an earlier and more accurate diagnosis of AF, enabling timely delivery of intervention and appropriate treatment strategies. However, whether this model can actually bring benefits to the subjects needs to be further studied. Moreover, we can establish similar LSs based on the peak of the PPG signal in theory. Subsequently, the generated graph can be fed into existing models to diagnose AF. This would make the algorithm much wider applicable.

## Limitations

There were several limitations to this study. First, our study used the CNN to establish a diagnosis model. This model is a black box that has poor interpretability. Explainable models are more popular in general, which can prevent some of the poor decisions in medicine that are caused by black box models [49]. Second, the research subject of the MS-AF dataset was collected from a primary care setting. However, we did not continuously include subjects due to interruptions caused by the COVID-19 pandemic, which caused selection bias for subjects. This is also the reason why the rate of AF in the MS-AF dataset was as high as 40.65%. Third, the number of patients with paroxysmal AF was only five patients in the MS-AF dataset, which is a low number and a low proportion of AF patients in the current study. This may affect the test results of the model. Fourth, limited by the types and number of arrhythmias in the database, the current model can only distinguish between non-AF, paroxysmal AF and persistent AF. In the classification of non-AF, it also includes other arrhythmias, such as atrial flutter. Compared to sinus rhythm, these conditions are associated with an increased risk of stroke or other complications. Hence, the presence of non-AF does not indicate that the subjects are completely free from underlying pathological conditions. With the accumulation of ECG data, we can develop machine learning diagnostic models for other arrhythmias to help general practitioners to identify more high-risk groups. Fifth, the database in this study may not fully capture the diversity of primary care population. More diverse and representative datasets from primary care settings are needed to validate and extend our findings. And we lacked a direct comparison with current standard-of-care methods and other machine learning algorithms within the same dataset.

## Conclusion

We developed a novel model for detecting AF episode based on a nonlinear LS and deep learning. The model had high accuracy and had application value in AF screening in primary care.

### Electronic supplementary material

Below is the link to the electronic supplementary material.

Supplementary Material 1

## Data Availability

The datasets used and/or analysed during the current study are available from the corresponding author on reasonable request.

## Abbreviations

AF: Atrial fibrillation
LS: Lorenz scattergram
CNN: Convolutional neural network
MIT-BIH AE: MIT-BIH Ambulatory Electrocardiograph
MS-AF: MOBILE-SCREEN-AF
Sen: Sensitivity
Spe: Specificity
Acc: Accuracy
PPV: Positive predictive value
NPV: Negative predictive value
+LR: Positive likelihood ratio
-LR: negative likelihood ratio
ROC: Receiver operating characteristic curve
AUC: Area under the curve
LSTM: Long short-term memory
