# Supplementary material for: Detection of atrial fibrillation using a nonlinear Lorenz Scattergram and deep learning in primary care
Source: BMC Prim Care. 2024 Jul 20;25:267. doi: 10.1186/s12875-024-02407-3 (PMC11265054; doi:10.1186/s12875-024-02407-3)
Supplement: Supplementary file 1 — Supplementary Material 1 [file 12875_2024_2407_MOESM1_ESM.docx]

***Supplementary Information***

**Detection of atrial fibrillation using a nonlinear Lorenz scattergram and deep learning in primary care**

Yi Yao^1,2,3^, Yu Jia^1,2,3^, Miaomiao Wu^1,2,3^, Songzhu Wang^1,2,3^, Haiqi Song^1,2,3^, Xiang Fang^1,2,3^, Xiaoyang Liao^1,2,3^, Dongze Li^4*^, Qian Zhao^1,2,3*^

^1^General Practice Ward/International Medical Center Ward, General Practice Medical Center, West China Hospital, Sichuan University, Chengdu, China

^2^Teaching&Research Section, General Practice Medical Center, West China Hospital, Sichuan University, Chengdu, China

^3^General Practice Medical Center and General Practice Research Institute, West China Hospital, Sichuan University, Chengdu, China

^4^Department of Emergency Medicine and Laboratory of Emergency Medicine, West China Hospital, Sichuan University, Chengdu, China.

***Correspondence:**

Dongze Li, MD, Department of Emergency Medicine and Laboratory of Emergency Medicine, West China Hospital, Sichuan University, Chengdu, China. Phone:+86 028 8542 2404. Email: dongze.li@ymail.com.

Qian Zhao, MD, General Practice Ward/International Medical Center Ward, General Practice Medical Center, West China Hospital, Sichuan University, Chengdu, China. Phone:+86 028 8542 1246. Email: 27355151@qq.com.

1. **The wearable ECG device.**


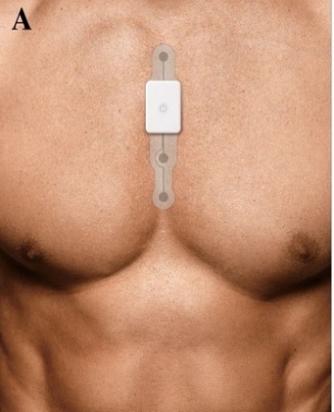


Appendix Figure 1. The single-lead wearable ECG device.

1. **The structure of convolutional neural network.**

The role of the convolutional layer is to extract image features. Combined with previous studies[1], the size of the convolution kernel selected in this study was 3 × 3. After evaluation, three artificial intelligence algorithm engineers agreed that 2-3 convolutional layers met the requirements, so the number of convolutional layers was set to 3. The stride of the sliding convolution kernel was set to 1[2]. This study used the leaky ReLU function as the activation function, which avoids neuron necrosis. Based on previous research, when the input value x was negative, the slope was set to 0.1[3]. The pooling layer was connected to the convolutional layer, which can reduce model complexity by reducing the data dimensions and the number of parameters of the model. Max pooling is one of the common pooling operations and was chosen in this study[4]. The pooling layer size in this study was set to 2 × 2[5]. The flattening layer was the one-dimensionalization of multidimensional input data, often used in transitioning from the convolutional layer to the fully connected layer. A dropout layer was added after the flattening layer in this study to avoid overfitting. The dropout layer randomly deactivates some neurons during training to avoid overfitting, and we selected a probability of deactivated neurons of 40% [2]. The role of the fully connected layer was to output the result. The node of the fully connected layer was connected to each node of the previous layer to synthesize the features extracted by the model.

Appendix Table 1. The structure of the convolutional neural network

| Layer | The output data structure of each layer |
| --- | --- |
| Convolutional 1 | (32，32，16) |
| Batch Normalization 1 | (32，32，16) |
| Max pooling 1 | (16，16，16) |
| Convolutional 2 | (16，16，32) |
| Batch Normalization 2 | (16，16，32) |
| Max pooling 2 | (8，8，32) |
| Convolutional 3 | (8，8，32) |
| Batch Normalization 3 | (8，8，32) |
| Max pooling 3 | (4，4，32) |
| Flattening Layer | (512) |
| Dropout Layer 1 | (512) |
| Fully Connected 1 | (32) |
| Dropout Layer 2 | (32) |
| Fully Connected 2 | (1) |

Reference

1. Kisohara M, Masuda Y, Yuda E, Ueda N, Hayano J. Optimal length of R-R interval segment window for Lorenz plot detection of paroxysmal atrial fibrillation by machine learning. Biomed Eng Online. 2020;19(1):49; doi: 10.1186/s12938-020-00795-y.

2. Gülcü A, Z K. Hyper-Parameter Selection in Convolutional Neural Networks Using Microcanonical Optimization Algorithm. IEEE Access. 2020;8:52528-40; doi: 10.1109/ACCESS.2020.2981141.

3. Jamart K, Xiong Z, Talou GM, Stiles MK, Zhao J: Two-Stage 2D CNN for Automatic Atrial Segmentation from LGE-MRIs. In: Statistical Atlases and Computational Models of the Heart Multi-Sequence CMR Segmentation, CRT-EPiggy and LV Full Quantification Challenges. Edited by Pop M, Sermesant M, Camara O, Zhuang X, Li S, Young A, et al. Cham: Springer International Publishing; 2020: 81-9.

4. Erdenebayar U, Kim H, Park JU, Kang D, Lee KJ. Automatic Prediction of Atrial Fibrillation Based on Convolutional Neural Network Using a Short-term Normal Electrocardiogram Signal. J Korean Med Sci. 2019;34(7):e64; doi: 10.3346/jkms.2019.34.e64.

5. Alzubaidi L, Zhang J, Humaidi AJ, Al-Dujaili A, Duan Y, Al-Shamma O, et al. Review of deep learning: concepts, CNN architectures, challenges, applications, future directions. J Big Data. 2021;8(1):53; doi: 10.1186/s40537-021-00444-8.

**3. The accuracy and loss value of the diagnostic model of each epoch in CNN training and validation.**


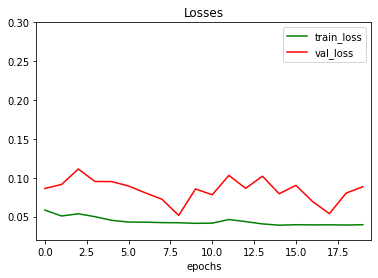


Appendix Figure 2. Loss function value of DM-AF in CNN training and internal validation set.

Epochs: the number of training cycles of the model. train_loss: the loss function value of the training set. val_loss: the loss function value of internal validation set.


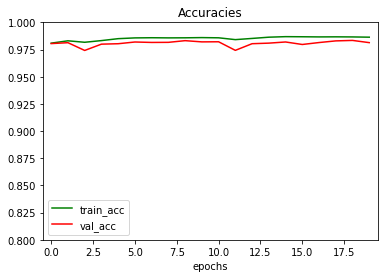


Appendix Figure 3. Accuracy of DM-AF in CNN training and internal validation set.

Epochs: the number of model training cycles. train_acc: the accuracy of training set. val_acc: the accuracy of internal validation set.

**4. Visual analysis of the convolution kernel of the convolutional layers.**

The image features of convolution kernel of each convolutional layers were showed in Appendix Figure 4, Appendix Figure 5 and Appendix Figure 6. In the figure, black represents a pixel value of 0, white represents a value of 1, and other different gray levels indicate values between 0 and 1.


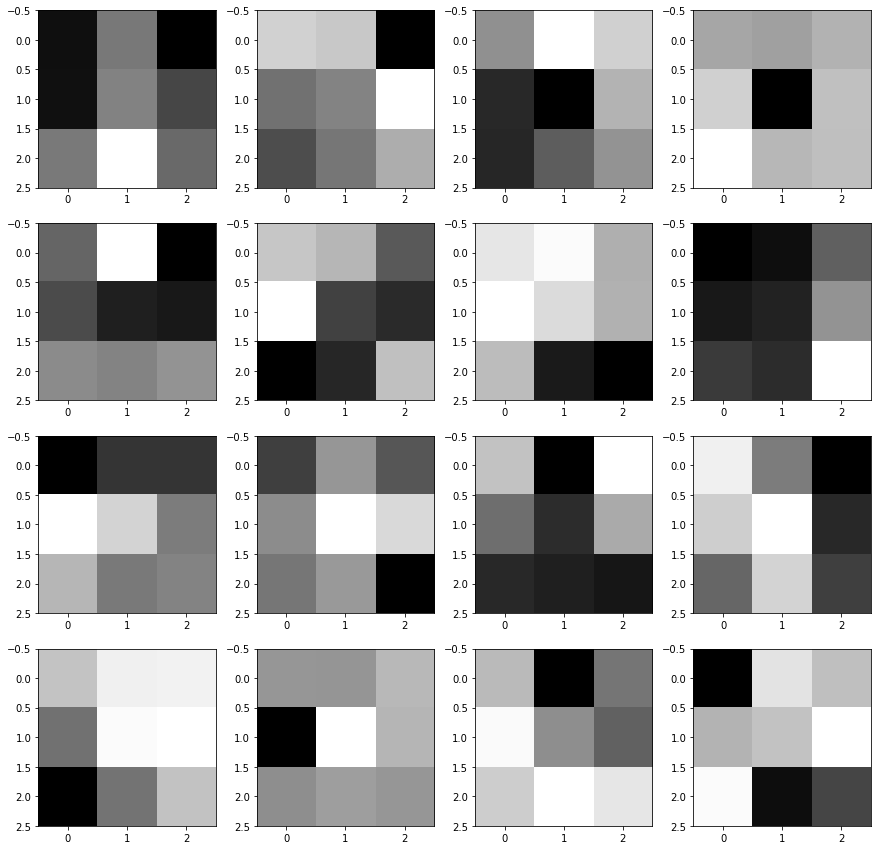


Appendix Figure 4. Image features of the convolution kernel of the first convolutional layer of the model


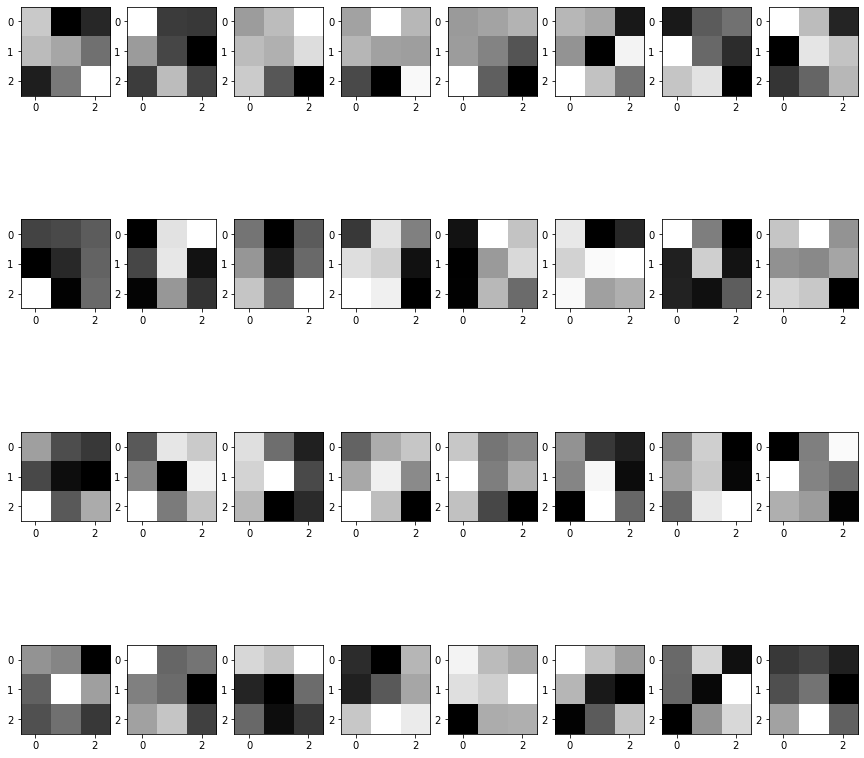


Appendix Figure 5. Image features of the convolution kernel of the second convolutional layer of the model


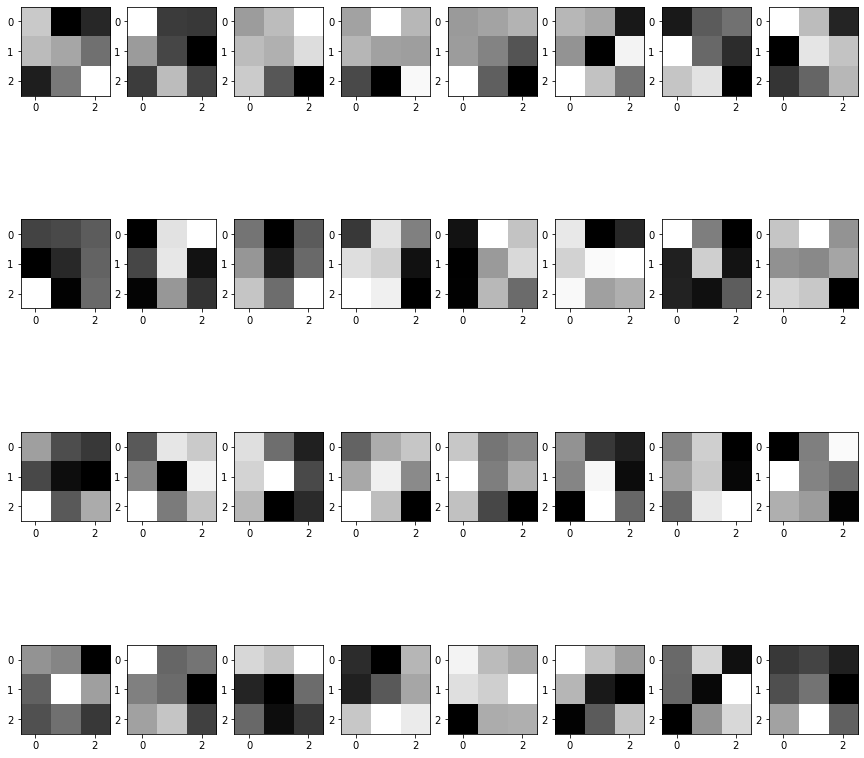


Appendix Figure 6. Image features of the convolution kernel of the third convolutional layer of the model
